# SNP detection and prediction of variability between chicken lines using genome resequencing of DNA pools

**DOI:** 10.1186/1471-2164-11-665

**Published:** 2010-11-25

**Authors:** Stefan Marklund, Örjan Carlborg

**Affiliations:** 1 Department of Animal Breeding and Genetics, Swedish University of Agricultural Sciences, Box 7023, SE-750 07 Uppsala, Sweden; 2 Linnaeus Centre for Bioinformatics, Uppsala University, BMC, Box 598, SE-751 24, Uppsala, Sweden

## Abstract

**Background:**

Next-generation sequencing technologies are widely used for detection of millions of Single Nucleotide Polymorphisms (SNPs) and also provide a means of assessing their variation. This information is useful for composing subsets of highly informative SNPs for region-specific or genome-wide analysis and to identify mutations regulating phenotypic differences within or between populations. In this study, we investigated the sensitivity of SNP detection and introduced the flanking SNPs value (FSV) as a novel measure for predicting SNP-variability using ~5X genome resequencing with ABI SOLID and DNA pools from two chicken lines divergently selected for juvenile bodyweight.

**Results:**

Genotyping with a 60 K SNP chip revealed polymorphisms within or between two divergently selected chicken lines for 31 363 SNPs, 48% of which were also detected using resequencing of DNA pools. SNP detection using resequencing was more powerful for positions with larger differences in allele frequency between the lines. About 50% of the SNPs with non-reference allele frequencies in the range 0.5-0.6 and 67% of those with frequencies > 0.9 could be detected. On average, ~3.7 SNPs/kb were detected by resequencing, with about 5% lower density on microchromosomes than on macrochromosomes. There was a positive correlation between the observed between-line SNP variation from the 60 K chip analysis and our proposed FSV score computed from the genome resequencing data. The strongest correlations on macrochromosomes and microchromosomes were observed when the FSV was calculated with total flanking regions of 62 kb (correlation 0.55) and 38 kb (correlation 0.45), respectively.

**Conclusions:**

Genome resequencing with limited coverage (~5X) using pooled DNA samples and three non-reference reads as a threshold for SNP detection, identified 50 - 67% of the 60 K SNPs with a non-reference allele frequency larger than 0.5. The SNP density was around 5% lower on the microchromosomes, most likely because of their higher gene content. Our proposed method to estimate the SNP variation (FSV) uses additional sequence information to better predict SNP informativity. The FSV scores showed higher correlations for SNPs with a larger difference in allele frequency between the populations. The correlation was strongest on macrochromosomes, probably due to a lower recombination rate.

## Background

Next generation sequencing technologies and SNP-chip genotyping with genome-wide coverage are affordable high-throughput genomics tools that are now being used in many research projects. Thus, large amounts of data are being quickly generated that will increasingly require novel methods for efficient data mining and analysis. In many genetic studies aimed at identifying genes determining phenotypic variation in species or populations, two phenotypically divergent groups or populations are compared to search for underlying mutations, genes and pathways. Typically, initial mapping of contributing loci is done using either genome-wide association studies (GWAS) or genome scans for quantitative trait loci (QTL). The initial mapping usually highlights a relatively large genomic region containing one or several genes with a phenotypic effect on the trait and is thus often followed by fine-mapping strategies using sample material where the causative mutation(s) are present on shorter haplotype blocks due to more generations of recombination [1,2]. Fine mapping can often be improved by adding more genetic markers, particularly if the haplotype blocks in the populations studied are shorter than the average distance between the original markers used for mapping. The new markers should be highly informative for the groups or populations being compared. Such markers are also crucial in marker-based breeding strategies such as generating introgression and congenic lines, where individuals should have different genotypes in regions of interest but homogenous genomic backgrounds [3,4]. The task of efficiently identifying suitable genetic markers is particularly challenging when there is considerable genetic variation within each line or group. This is usually the situation with outbred domestic animals, which may show considerable phenotypic differences between strains or breeds, but also be highly genetically variable within breeds.

Here, we have evaluated the sensitivity of informative SNP detection using genome resequencing. Pooled samples from two chicken lines that were divergently selected for 41 generations [5] have been resequenced with 5X depth coverage. The lines display a nine-fold difference in body weight at 56 days of age and have previously been used for QTL mapping of growth related traits [6,7]. We present a computational method where the information from flanking SNPs is used as an indicator of genotypic divergence between resequenced populations and compare the results from this method to those obtained by individual genotyping with the Illumina 60 K chicken SNP chip.

## Results

### 60 K SNP chip genotyping

**Figure 1:**
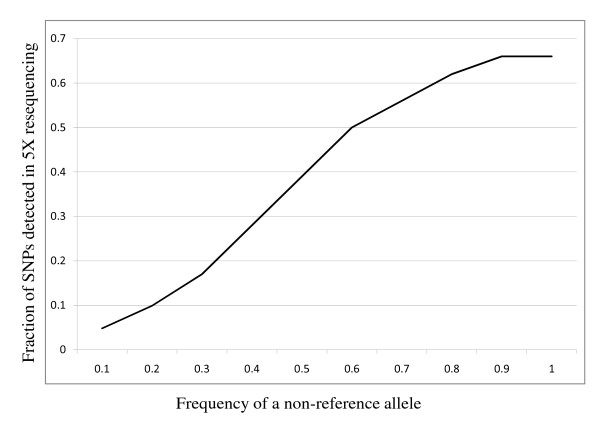
**Sensitivity of SNP detection with 5X genome resequencing**. Fraction of SNPs detected in ~5X genome resequencing for SNPs classified based on the 60 K SNP frequency of a non-reference allele.

The 60 K SNP chip genotyping of 20 individuals from each of the high and low lines sampled at generation 41 assayed 53 313 SNPs located on autosomes 1 to 28. A total of 51 894 SNPs (97%) had a call frequency > 0.95. Polymorphism within or between the high and low lines was detected for 31 363 SNPs (59%) and of these, 15 193 (48%) were also detected with the ABI SOLID resequencing. As expected, the fraction of 60 K SNPs detected with the resequencing increased with the frequency of the non-reference allele in the population represented in the DNA pool (Figure 1).

### Genome resequencing

The resequencing of chicken autosomes 1- 28 resulted in ~915 Mbp of consensus sequence (excluding Ns indicating gaps) from each of the lines, which corresponds to ~99.7% of the Red Jungle Fowl reference sequence from these chromosomes according to the May 2006 assembly of the chicken genome [8]. The depth coverage was 5.19X and 5.53X for the high and low line, respectively, as recently reported [9]. This revealed a total of 3 342 812 SNPs, which corresponds to around 3.7 SNPs/kb. The number and fraction of SNPs detected, classified according to their variation within line against the RJF reference, are given in Table 1. The SNP density varied slightly between macrochromosomes (GGA1-5, ~3.6 SNPs/kb), chromosomes of intermediate length (GGA6-10, ~3.9 SNPs/kb) and microchromosomes (GGA11-28, ~3.4 SNPs/kb). The observed SNP detection rate with 5X genome resequencing of DNA pools for different non-reference allele frequency classes in the represented populations are shown in Figure 1. On average, nearly six reads covered each detected SNP, with approximately 1% more coverage on macrochromosomes than on microchromosomes.

**Table 1:** SNP variation within line observed with the 5X genome resequencing.

SNP variation	No. of SNPs	Fraction of SNPs (%)
All reads from both lines differ from the reference	719 288	22

All reads from one line differ from the reference	1 338 437	40

One line biallelic	1 175 291	35

Both lines biallelic	109 796	3

Counts and fraction of the 3 342 812 SNPs detected in genome resequencing based on their variation within line and against the RJF reference.

### Correlation of allele frequency differences between lines from the 60 K SNP genotyping and the flanking SNPs value from resequencing

**Figure 2:**
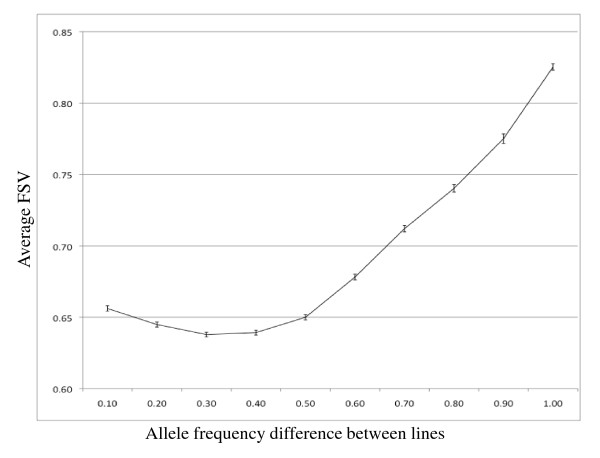
**Flanking SNPs values (FSV) for different levels of observed SNP variation between lines**. Average flanking SNPs values (FSV; ± SE) for SNPs classified based on allele frequency difference between lines according to 60 K SNP chip genotyping.

**Figure 3:**
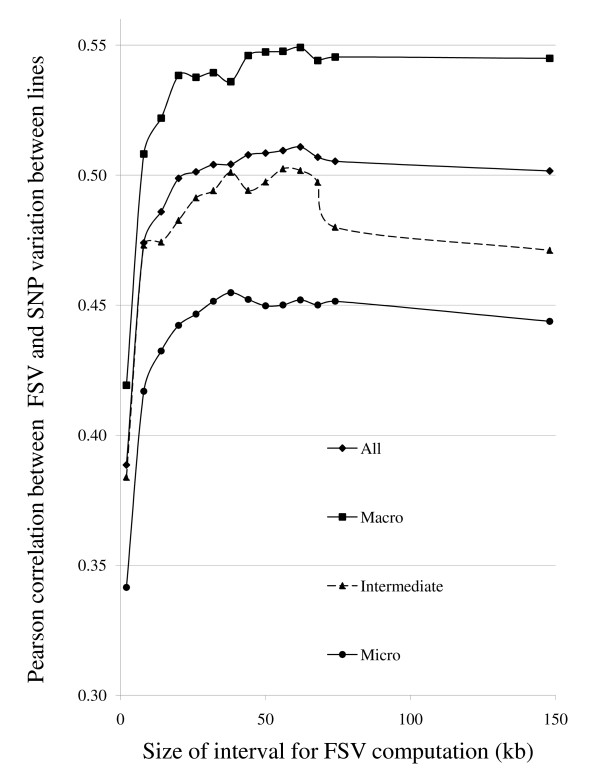
**Correlation between flanking SNPs value (FSV) and SNP variation between lines**. Pearson product-moment correlations between FSV and 60 K SNP allele frequency difference between lines for different FSV computation intervals and chromosome size classes. SNPs with 0.4 or larger allele frequency difference between lines were included.

The overall correlation between the 60 K SNP allele frequency difference between lines and the flanking SNPs value (FSV) was moderate (Pearson r = 0.36, P < 10^-15^) for the FSV interval with the strongest correlation (62 kb). The correlation was, however, highly dependent on the difference in allele frequency between the lines. There was no correlation for SNPs with low variation between lines and a clear correlation for SNPs with more variation between lines (Figure 2). For SNPs with an allele frequency difference between lines > 0.4, the genome-wide (GGA1-28) correlation between the two measures was highest when the interval used for FSV computation was 62 kb (Pearson r = 0.51; P < 10^-15^). As shown in Figure 3, the degree of correlation, as well as the size of FSV interval that gave the highest correlation, varied between chromosome size classes. The macrochromosomes (GGA1-5), showed maximum correlation with a 62 kb FSV interval (Pearson r = 0.55, P < 10^-15^), whereas for the intermediate chromosomes (GGA6-10) and the microchromosomes (GGA11-28) the highest correlations were with 56 kb (Pearson r = 0.50; P < 10^-15^) and 38 kb intervals (Pearson r = 0.45; P < 10^-15^), respectively (Figure 3).

## Discussion

In this study, we have compared SNP detection rates in resequencing of pooled samples from two genetically divergent chicken lines with allele frequency estimates using 60 K SNP genotyping of individual samples. Using a SNP detection threshold of three non-reference reads for each pool, a total of approximately 3.7 SNPs/kb were found in the resequencing data. This is less than the around 5 SNPs/kb found in previous chicken comparisons [10]. Furthermore, less than 50% of the 60 K SNPs that showed variation within or between the chicken lines were detected in the resequencing data. The reason for this low detection rate is the limited depth coverage (about 5X) combined with a low non-reference allele frequency among the undetected SNPs. On the other hand, when there is a large allele frequency difference between lines, one line will have a high frequency of a non-reference allele, which increases the probability to reach over the SNP detection threshold of three non-reference reads in resequencing (Figure 1). In many studies, the most interesting SNPs will be those where there is a high allele-frequency difference or fixation for alternative alleles in the studied populations as these are putative causative mutations or represent opposite selective sweeps. Here we have reported, however, that resequencing of pools of individuals with approximately 5X depth coverage failed to identify 33% of the SNPs that were fixed for alternative alleles in the two lines according to the SNP chip data (Figure 1). Thus, a higher depth coverage in genome resequencing and additional resequencing of targeted regions should be considered to increase the detection sensitivity.

We have proposed a new measure for identifying informative SNPs from low coverage resequencing data, which takes into account variation in flanking SNPs (FSV). We have also demonstrated a positive correlation between FSV and the difference in allele frequency between lines from the 60 K SNP chip data. The correlation was strongest on the macrochromosomes when FSV was calculated with a 62 kb interval. For the microchromosomes the optimal interval size was considerably shorter (38 kb) and the correlation weaker, probably due to a higher recombination rate and/or a higher mutation rate [11,12]. However, despite a presumed higher mutation rate on microchromosomes, the overall density of SNPs detected in resequencing was approximately 5% lower on the microchromosomes than on macrochromosomes. This is probably due to a considerably higher gene density on the microchromosomes and consequently a larger proportion of conserved sequence under purifying selection [13]. The optimal FSV interval size with the highest correlation between FSV and SNP variation between lines may approximately correspond to the average LD block size in these populations.

No correlation was observed between FSV and difference in allele frequency between lines that were lower than 0.4. A plausible explanation is that the FSV is most affected by random sampling effects for the few sequencing reads covering the SNP, when the difference in allele frequencies is small. For example, if both lines show a high frequency for a non-reference allele there will be many cases where only one of the lines reaches over the SNP detection level, whereas the other line will be assumed to be fixed for the RJF allele. This would incorrectly increase the FSV and lower the correlation with the true SNP variation between lines. In other words, most random sampling effects among sequencing reads from a pool of DNA samples would increase the FSV and thereby reduce the correlation with SNP variation between lines if that variation is small. Another possible explanation is that large difference in allele frequencies between the two lines studied at a biallelic locus includes less sensitivity to such random sampling effects, because of one predominant allele in each line that is relatively rare or absent in the other line.

In summary, our results show that the depth coverage and pooling of samples limits SNP detection sensitivity using resequencing. The described measure, however, makes better use of available sequence information than studies using only individual SNP positions and can thereby provide a valuable indication of the SNP variation-between resequenced populations, even when the depth coverage is quite low and pooled samples are sequenced. This could, for example, facilitate the selection of the most informative SNPs for a genotyping panel among millions of putative candidates detected in resequencing. In selecting SNPs it is also important to consider how many reads covered the SNP position and the individual SNP score indicating the fraction of reads in agreement with the reference. When there exist a very large number of SNPs to choose from it would be possible to stringently select SNPs with high FSV, large number of covering reads and a high proportion of non-reference reads.

## Conclusions

Genomic resequencing with limited coverage (~5X) using pooled DNA samples can be used to detect the majority of SNPs with an allele frequency difference larger than 0.5 between the pools. For any detected SNP, computation of the variation among flanking SNPs (FSV) based on the resequencing read scoring, can be used for estimating of the degree of polymorphism between the populations. This correlation is more accurate for chicken macrochromosomes than for microchromosomes, probably due to a higher recombination rate and more haplotype variation on the microchromosomes.

## Methods

### Animals, resequenced genomic DNA pools and samples for 60 K SNP chip genotyping

The Virginia high and low body weight selected chicken lines were studied. The Virginia lines are chicken resource populations, which have been bred in a long-term, divergent selection experiment for body weight at 56 days of age. After 38 generations, the lines showed a near nine-fold difference in bodyweight at the selection age and large differences in a large number of correlated traits including fat deposition, appetite and immune response [5]. In this study, we used chickens from generation 41, which had previously been used as founders of an F2 intercross used for QTL mapping [6,7]. Twenty individuals from each of the high and low lines were genotyped with the 60 K SNP Illumina iSelect chicken array developed by USDA Chicken GWMAS Consortium, Cobb Vantress, and Hendrix Genetics. Resequencing was carried out on one DNA pool from each line. Each pool included seven males and four females and all samples in these pools except one low line sample were also among the 60 K genotyped samples. The protocol for animals and collection of blood samples was approved by the Virginia Tech Animal Care and Use Committee (IACUC).

### Genome resequencing and SNP identification

Genome resequencing was carried out using the Applied Biosystems SOLID v2 technology according to the manufacturer's protocols, with one fragment library from each pool and 35 bases per read. The reads were aligned to the Red Jungle Fowl reference genome sequence [8] as described elsewhere [9] using at least three reads of a non-reference allele in the sequenced DNA pool, as a threshold for SNP calling.

### Test for correlation between 60 K SNP allele frequencies and the flanking SNP detection pattern from resequencing

With resequencing SNP calling using the Corona Lite pipeline from Life Technologies, each detected SNP has a score indicating the proportion of reads that are in agreement with the Red Junglefowl (RJF) reference sequence. With very high depth coverage, this score could be used as an indicator of the allele frequency difference between lines. With low sequence coverage, however, the score results could be misleading as many SNPs that actually show variation within a line might appear to be fixed.

To make better use of the available information from the resequencing, we propose a new method to estimate the informativity of a SNP based on the variation in SNPs flanking the tested position - the Flanking SNPs Value (FSV). This value is calculated as follows:

$$FSV=\frac{\left(\sum_{i=1}^{N_{H}}\left|S_{c_{i}}^{H}-S_{c_{i}}^{L}\right|\right)}{N_{H}}\times \frac{\left(\sum_{j=1}^{N_{L}}\left|S_{d_{j}}^{L}-S_{d_{j}}^{H}\right|\right)}{N_{L}}$$

where $S_{c_{i}}^{H}$ and $S_{c_{i}}^{L}$ are the resequencing scores in the high and low line, respectively, for SNP *i *detected in the high line at position coordinate *c*. Likewise, $S_{d_{j}}^{L}$ and $S_{d_{j}}^{H}$ are the corresponding scores for SNP *j *detected in the low line at position coordinate *d*.

N_H _and N_L _are the total number of high line SNPs and low line SNPs, respectively, scored within the flanking regions.

To evaluate how well FSV predicted SNP variability between lines from resequencing data, we studied the correlation between FSV and SNP variation between lines measured using SNP chip genotyping. All autosomal SNPs included on the 60 K chip that showed variation within or between the high and low lines were used. We included the chicken autosomes 1 to 28 because they were fully represented in both the 60 K and genome resequencing datasets. Most SNPs in the resequencing were detected in only one of the lines and then we assumed fixation for the reference Red Junglefowl (RJF) allele in the other line. SNPs where none of the reads in either line had the RJF allele were excluded from the calculation. Intervals with a SNP density higher than 1 SNP/kb for each line were considered to show sufficient information to be included in the correlation test. Previous estimates of 5 SNPs/kb for most chicken line comparisons [10] suggest that even the most highly conserved regions can be expected to reach above this threshold unless the depth coverage is particularly poor. Scripts in Python [[14], Additional file 1] were used to compute FSVs of the SNPs of interest for flanking regions totalling 2, 8, 14, 20, 26, 32, 38, 44, 50, 56, 62, 68, 74 and 128 kb. The R program package [15] was used to test the Pearson's product-moment correlation between FSVs and the observed differences in allele frequency from the SNP chip data.

## Authors' contributions

SM developed the Python pipeline for analysis of the 60 K and ABI SOLID SNP detection data, analysed the data and did the major part of the manuscript preparation.

SM and ÖC planned the work and regularly discussed the study including revisions of the manuscript.

## Supplementary Material

Additional file 1 **Python scripts used for computation of 60 K SNP allele frequencies and FSV**. Click here for file
